# Intelligent Decision-Making of Scheduling for Dynamic Permutation Flowshop via Deep Reinforcement Learning

**DOI:** 10.3390/s21031019

**Published:** 2021-02-02

**Authors:** Shengluo Yang, Zhigang Xu, Junyi Wang

**Affiliations:** 1 Shenyang Institute of Automation, Chinese Academy of Sciences, Shenyang 110016, China; yangshengluo@sia.cn (S.Y.); jywang@sia.cn (J.W.); 2 Institutes for Robotics and Intelligent Manufacturing, Chinese Academy of Sciences, Shenyang 110169, China; 3 University of Chinese Academy of Sciences, Beijing 100049, China

**Keywords:** permutation flowshop scheduling problem, deep reinforcement learning, actor-critic, dynamic scheduling, real-time scheduling, new job arrival, tardiness cost

## Abstract

Dynamic scheduling problems have been receiving increasing attention in recent years due to their practical implications. To realize real-time and the intelligent decision-making of dynamic scheduling, we studied dynamic permutation flowshop scheduling problem (PFSP) with new job arrival using deep reinforcement learning (DRL). A system architecture for solving dynamic PFSP using DRL is proposed, and the mathematical model to minimize total tardiness cost is established. Additionally, the intelligent scheduling system based on DRL is modeled, with state features, actions, and reward designed. Moreover, the advantage actor-critic (A2C) algorithm is adapted to train the scheduling agent. The learning curve indicates that the scheduling agent learned to generate better solutions efficiently during training. Extensive experiments are carried out to compare the A2C-based scheduling agent with every single action, other DRL algorithms, and meta-heuristics. The results show the well performance of the A2C-based scheduling agent considering solution quality, CPU times, and generalization. Notably, the trained agent generates a scheduling action only in 2.16 ms on average, which is almost instantaneous and can be used for real-time scheduling. Our work can help to build a self-learning, real-time optimizing, and intelligent decision-making scheduling system.

## 1. Introduction

Production scheduling is one of the important issues to consider for a production workshop. Since flowshop is one of the most widely used workshops, the permutation flowshop scheduling problem (PFSP) has received numerous studies during the past several decades. According to a review by Fernandez-Viagas et al. [1], hundreds of heuristic and metaheuristic methods have been proposed to solve the PFSP over the past 60 years. Studies of PFSP have a significant impact on the scheduling field. The permutation criteria in PFSP restricts that the job sequence in the first machine is maintained for all successive machines, i.e., the job sequence is the same for all machines in the flowshop. The PFSP has been proved non-deterministic polynomial-time hard (NP-hard) when the number of machines is more than two [1]. To solve the NP-hard problem, many hybrid meta-heuristics have been proposed [2,3]. For the PFSP, its objective is to find a good job sequence to minimize makespan [4,5,6], flow time [7,8,9,10], tardiness [11,12,13,14], multiple objective [15,16,17,18,19,20], etc. 

Given the NP-hard of PFSP, many meta-heuristics, such as genetic algorithm (GA) [21,22,23,24], particle swarm optimization (PSO) [25,26,27], and iterated greedy (IG) algorithm [21,22,23,24], have been proposed to solve the PFSP. Generally, the PFSP is regarded as a statistic scheduling problem. However, in a real production environment, the workshop is dynamic with real-time events occurring, such as dynamic order arrival, machine breakdowns, rush order insertion, etc. Thus, dynamic scheduling has more practical implications for a real workshop. Several dynamic characteristics have been considered, such as dynamic job/order arrival [28,29,30,31], stochastic processing time [32,33,34], machine breakdown [35], process interruptions [36,37], etc.

Among these dynamic characteristics, new job arrival has been recently receiving arising attention. In this problem, jobs arrive at the production system randomly rather than at time zero, as generally assumed for statistic PFSP. A rescheduling is needed when a new job arrives. This problem is described as a real-time scheduling problem in some literature [25,38,39,40].

Several researchers have studied the dynamic PFSP with new job arrival using heuristics and meta-heuristics. Rahman, Sarker and Essam [38] investigated the PFSP with real-time order arrival and used GA to repeatedly re-optimize the solution as each new order arrives. Liu et al. [41] proposed a scheduling strategy by integrating match-up and real-time strategy and provided eleven new heuristics with ten existing and one new priority rule. Li et al. [42] solved the flow shop scheduling problem with new job arrival using the constructive heuristic method. The heuristic method successively puts each job in the current best position and reinserts several jobs based on weight calculations. Liu et al. [43] studied the dynamic PFSP with new job arrival using an improved IG, which is equipped with a novel repair method and new enhanced destruction and reconstruction. 

With the advance of artificial intelligence (AI), DRL and reinforcement learning (RL) have also been used to optimize scheduling problems. The DRL method trains a scheduling agent to choose the best job to process next based on current production status. Since a scheduling problem can be regarded as a sequential decision problem, it is suitable to be solved by DRL [44]. Many scheduling problems, such as flowshop scheduling problem (FSP), job shop scheduling problem (JSP), assembly shop scheduling problem (ASP), parallel machine scheduling problem, etc., have been solved by DRL or RL. For FSP, Zhang et al. [45] studied the FSP using an online TD algorithm to minimize makespan. For JSP, several studies have been carried out both for statistic and dynamic JSP. Lin et al. [46] studied the JSP under an edge computing framework and used a multiclass deep Q network (DQN) to generate scheduling decisions for multiple edge devices. Liu et al. [47] solved the JPS using an actor-critic algorithm. Zhang et al. [48] used the DRL to automatically learn priority dispatching rule for JSP. They exploited the disjunctive graph representation of JSP and proposed a Graph-Neural-Network-based scheme to embed sates. Han and Yang [49] studied the adaptive JSP using dueling double DQN. Luo [44] studied the dynamic JSP with new job insertions to minimize total tardiness using DRL. A DQN is used to select appropriate dispatching rules at each rescheduling point, and seven generic state features are extracted to represent production status. For other types of workshops, Wang et al. [50] investigated the adaptive scheduling for assembly job shop with uncertain assembly times using dual Q-learning algorithm, which contains top and bottom level Q-learning methods. Shiue, Lee and Su [39] studied a RL-based real-time scheduling problem using multiple dispatching rules strategy to respond to changes in a manufacturing system. Shiue et al. [51] studied the dynamic scheduling of a flexible manufacturing system and semiconductor wafer fabrication using RL. Zhang et al. [52] studied the scheduling of unreliable parallel machines to minimize mean weighted tardiness using RL.

In addition to the DRL, other AI approaches, such as machine learning, deep learning, and a combination of machine learning and meta-heuristics, are also used to solve scheduling problems in recent years. Jun and Lee [53] addressed the dynamic single-machine scheduling problem to minimize total weighted tardiness by learning dispatching rules from schedules. They proposed a decision-tree-based machine learning method to extract dispatching rules from existing schedules. Wu et al. [54] used deep learning to solve unreliable machines’ dynamic dispatching in re-entrant production systems. They combine a deep neural network (DNN) and Markov decision processes (MDP) to assign different priorities to job groups to minimize cycle time or maximize throughput. Li et al. [55] studied the flexible job-shop scheduling problem (FJSP) with sequence-dependent setup times and limited dual resources using machine learning and meta-heuristics. A hybrid meta-heuristic is proposed to solve the FJSP, and the machine learning classification model is trained to identify rescheduling patterns. Chen et al. [56] used RL to control key parameters of GA during evolution in FJSP.

From the above literature review, we can know that the dynamic PFSP with new job arrival has become an active topic in recent years and has been studied using several meta-heuristics. Recently, DRL and other AI techniques serve as a new approach to solve scheduling problems. However, the dynamic PFSP with new job arrival and total tardiness cost criteria has not been solved by DRL. Since the modeling of dynamic flowshop is different from those of other workshops, it is necessary to propose a DRL-based modeling method for dynamic PFSP. In addition, the DRL-based approaches are not compared with traditional meta-heuristics in most literature. It is unclear whether the DRL algorithms outperform traditional meta-heuristics considering solution quality and CPU times. 

This paper studies the dynamic PFSP with new job arrival to minimize total tardiness cost using DRL. Our study aims to realize real-time optimization and intelligent decision-making of scheduling for dynamic flowshop using DRL. The procedure of solving dynamic PFSP using DRL is illustrated, and the mathematical model is formulated. The scheduling agent is then modeled by designing state features, actions, and reward for the DRL agent. The A2C algorithm is adapted to train the scheduling agent to choose appropriate actions in different production status. The results show the well performance of the A2C-based scheduling agent compared with other DRL algorithms and meta-heuristics.

In particular, the contributions of this paper are as follows.

(1) To the best of our knowledge, this is the first attempt to solve the dynamic PFSP with new job arrival to minimize total tardiness cost using DRL. Our work can fill the research gap in solving dynamic PFSP by DRL.
(2) An intelligent decision-making scheduling system based on DRL is designed and modeled. The system architecture of intelligent scheduling is proposed. States features, actions and reward for the scheduling agent and system are designed.
(3) Our work shows the DRL-based scheduling method outperforms traditional meta-heuristics (IG and GA) in solution quantity and CPU times by a large margin for dynamic FPSP.

## 2. Problem Description and Mathematical Model

### 2.1. Problem Description

**Figure 1:**
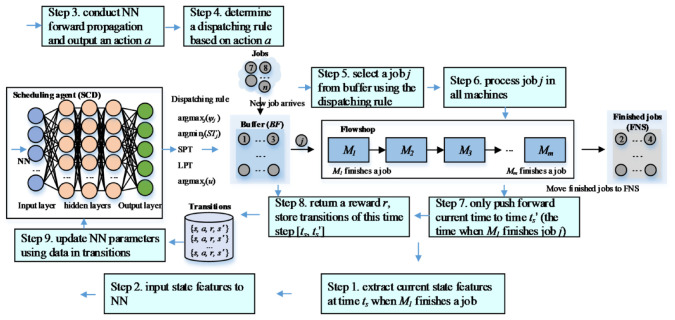
The system architecture of solving dynamic PFSP using DRL.

In this section, the system architecture of solving dynamic PFSP using DRL is proposed. As shown in Figure 1, a new job arrives at the system randomly. If the job cannot be processed immediately, the job will be stored in a buffer *BF*. The flowshop needs to determine which job to process next using scheduling agent SCD when a job is finished in *M*_1_. The current state features, including job information and production status, are input to SCD to generate a scheduling action. Since for PFSP, the job sequence is maintained for all machines. Only *M*_1_ needs to determine which job will be processed next. Thus, for step 7 in Figure 1, the system time is only pushed to time *t_s_*_’_ (when *M*_1_ finishes job *j*) to determine which job will be processed next in *M*_1_ using SCD. At each decision point *t_s_*_’_, the jobs finished in all machines are moved to the finished jobs area, denoted as FNS. 

### 2.2. Mathematical Model

The mathematical model is established to minimize the total tardiness cost of all jobs arriving at the system. Some notations are listed as follows.

Notations:

*j*: index of jobs, *j* = 1, 2, …, *n*
*i*: index of machines, *i* = 1, 2, …, *m*
*t_ij_*: processing time of job *j* on machine *i*, *i* = 1, 2, …, *m*, *j* = 1, 2, …, *n*
*C_ij_*: completion time of job *j* on machine *i*, *i* = 1, 2, …, *m*, *j* = 1, 2, …, *n*
*C_j_*: completion time of job *j*, *j* = 1, 2, …, *n*
*d_j_*: due date of job *j*, *j* = 1, 2, …, *n*
*CP*: an indicator of completion time of all jobs
TF: tardiness factor
RDD: the relative range of due dates
$\alpha_{j}$: unit (per second) tardiness cost of job *j*
*AT_j_*: arrival time of job *j*

Based on the notations above and some related formulation works [57,58,59], the objective function is formulated as Equation (1).

(1) $$\mathrm{Minimize}\;\sum_{j=1}^{n}\alpha_{j}\times \max\{0,C_{j}-d_{j}\}$$

Subject to:

(2) $$d_{j}=[CP(1-\mathrm{TF}-\frac{\mathrm{RDD}}{2}),\;CP(1-\mathrm{TF}+\frac{\mathrm{RDD}}{2})],\;j=1,2,\ldots ,n$$

(3) $$CP=\frac{1}{m}\times \sum_{j=1}^{n}\sum_{i=1}^{m}t_{ij}$$

(4) $$C_{j}=C_{ij},\;i=m,\;\forall j$$

(5) $$C_{ij}\geq \max\{C_{(i-1)j},C_{i(j-1)}\}+t_{ij},\;\forall i,j$$

(6) $$C_{1j}-t_{1j}\geq AT_{j},\;\forall j$$

(7) $$C_{0j}=0,\;\forall j$$

(8) $$C_{i0}=0,\;\forall i$$

According to [58,60,61], the *d_j_* is set by constraint (2). The *d_j_* follows a uniform distribution and is controlled by *CP*, TF, and RDD. TF and RDD are constant and are set to 0.5 and 0.5 [58,60]. *CP*, is calculated by Equation (3). Equation (4) defines the completion time of a job in the system. Equation (5) gives the completion time of a job on a machine. Equation (6) ensures that a job can be processed by the first machine only after the job arrives at the system. Equations (7) and (8) provide some initial values of completion time *C_ij_*. 

## 3. Modelling of the Intelligent Scheduling System

This section models the intelligent scheduling system by designing state features, actions, and reward for the scheduling agent and workshop environment. Whenever *M*_1_ finishes a job, the scheduling agent SCD generates a scheduling action based on current state features, and a job is selected based on the scheduling action and processed on *M*_1_. The workshop environment returns a reward for this scheduling action to update SCD parameters. The reward, state features, and actions are designed as follows.

### 3.1. Reward

Since most literature on using DRL to solve the scheduling problems are aimed to minimize makespan. These reward functions cannot be used to minimize the total tardiness cost. We designed a new reward function for the total tardiness cost criteria in this section.

Recall that the aim of solving PFSP is to find out a job sequence with minimized total tardiness cost. The scheduling agent SCD should learn to choose an appropriate job at every decision point so that the total tardiness cost is minimized after all jobs are finished. Each action the SCD taken should make the total tardiness cost increases as little as possible. Given that DRL is designed to maximize the cumulated reward obtained at each step, the reward in this problem is defined as the inverse of unit newly added total tardiness cost of unfinished jobs in the system during this time step. The newly added tardiness cost comes from work-in-progress (*WIP*) and jobs in the buffer (*BF*). Therefore, the reward of time step [*t_s_*, *t_s_*_′_] is calculated by Equation (9).

(9) $$r=-\frac{1}{t_{s^{\prime }}-t_{s}}(tp_{BF}+tp_{WIP})$$

where *tp_WIP_*, *tp_BF_* denote newly added tardiness penalty cost from *WIP* and jobs in *BF*, respectively, during the current time step [*t_s_*, *t_s_*_′_]. *tp_BF_*, *tp_WIP_* are calculated as follows.

(10) $$tp_{BF}=\sum_{j=1}^{n_{BF}}z_{js^{\prime }}\alpha_{j}[t_{s^{\prime }}-\max(t_{s},d_{j})],\;j\in BF$$

(11) $$tp_{WIP}=\sum_{j=1}^{n_{{}_{WIP}}}z_{js^{\prime }}\alpha_{j}[t_{js^{\prime }}-\max(t_{s},d_{j})],\;j\in WIP$$

where *n_BF_* denotes the number of jobs in *BF*, *n_WIP_* denotes the number of *WIP*. *z_js_*_′_, defined by Equation (12), indicates whether job *j* is overdue at *t_s_*_′_, the end time of this step. Only the overdue jobs generate tardiness cost. *t_js_*_′_, calculated by Equation (13), denotes the actual end time for job *j* compared with *t_s_*_′_. If job *j* is completed, i.e., finished by all machines, before *t_s_*_′_, the time step for calculating tardiness cost is [*t_s_*, *C_j_*] rather than [*t_s_*, *t_s_*_′_].

(12) $$z_{j_{s^{\prime }}}=\left\{\begin{array}{cc} 1, & d_{j}<t_{s^{\prime }}\\ 0, & \mathrm{else} \end{array}\right.$$

(13) $$t_{js^{\prime }}=\left\{\begin{array}{cc} C_{j}, & \;\mathrm{if}\;\mathrm{job}\;j\;\mathrm{has}\;\mathrm{been}\;\mathrm{completed}\;\mathrm{at}\;t_{s^{\prime }}\\ t_{s^{\prime }}, & \mathrm{else} \end{array}\right.$$

### 3.2. State Features

The scheduling agent SCD determines a scheduling action based on current state features. State features should fully and efficiently reflect current job information and production status. Specifically, state features should provide sufficient information for selecting an action at each rescheduling point. The state features in most existing literature are designed to solve the JSP to minimize the makespan. These features are not applicable for our studied problem. Thus, we designed five state features for the PFSP under the total tardiness cost criteria, considering both jobs in *BF* and current production status. Unlike the existing literature, we calculated four statistic characteristics, maximum, minimum, average, and standard deviation, for several state features rather than only providing the average value to reflect each feature’s characteristics better.

The five state features, *ft*_1_, *ft*_2_, …, *ft*_5_, are defined as follows.

(1) $ft_{1}=\{\psi_{j}\},\;j\in BF$. Current unit tardiness cost of each job in *BF*.
  where $\psi_{j}$ is the unit tardiness cost generated by job *j* at present and is calculated as follows.
  (14) $$\psi_{j}=\left\{\begin{array}{rr} \alpha_{j}, & \mathrm{if}\;\mathrm{job}\;j\;\mathrm{overdues}\;\mathrm{at}\;\mathrm{current}\;\mathrm{time}\\ 0, & \mathrm{else} \end{array}\right.$$
(2) $ft_{2}=\{ST_{j}\},\;j\in BF$. Safe time of each job in *BF*.
  where *ST_j_* is determined by Equation (15). *ST_j_* reflects how much time will be left before the due date *d_j_* when job *j* is finished, if job *j* begins to be processed at present time *t_c_*.
  (15) $$ST_{j}=d_{j}-\sum_{i=1}^{m}t_{ij}-t_{c}$$
(3) $ft_{3}=\{\sum_{i=1}^{m}t_{ij}\},\;j\in BF$. Total processing times in all machines for each job in *BF*.
(4) $ft_{4}=\{{u_{j}}^{\prime }\},\;j\in BF$. The estimated utilization rate of each job in *BF*. *u_j_′* is the estimated utilization rate of job *j* and is calculated by Equation (16). Each job in *BF* is assumed to be processed under the present production status. The *u_j_′* is calculated based on the waiting times *WT_ij_′* of all machines when job *j* is processed.
  (16) $${u_{j}}^{\prime }=1-(\sum_{i=2}^{m}W{T_{ij}}^{\prime }/\sum_{i=1}^{m}t_{ij}),\;j\in BF$$
  (17) $$W{T_{ij}}^{\prime }=\max(0,\;C_{i(j-1)}-C_{(i-1)j}),i=2,3,\ldots ,m,j\in BF$$
  where *WT_ij_*′ is the waiting time of job *j* on machine *i*. *WT_ij_*′ generates when job *j* is finished on machine *i* − 1, but cannot be processed immediately on machine *i* because machine *i* has not finished its current job *j* − 1. Note that the first machine *M*_1_ does not have a waiting time because *M*_1_ is always idle at a decision point.
(5) $ft_{5}=n_{BF}$. The number of jobs in *BF* at present.

For the first four state features, which are array, four statistic characteristics, maximum, minimum, average, and standard deviation, are calculated. Thus, the total dimensions for state features are 4 × 4 + 1 = 17. In addition, all features are normalized to facilitate the learning process.

### 3.3. Actions

Each action corresponds to a single dispatching rule (SDR) for selecting a candidate job to be processed in *M*_1_ from *BF*. The action space should provide sufficient and effective dispatching strategies under different production status. To lower the learning difficulty, the action space should not be too large. We designed five actions (*a*_1_–*a*_5_) for SCD, considering different production status and well-known dispatching rules.

(1) Select the job *j*, which has the minimum current unit tardiness cost $\psi_{j}$

Where $\psi_{j}$ is calculated by Equation (14).

$$a_{1}=\arg\max_{j}\left(\psi_{j}\right),\;j\in BF$$

*a*_1_ is apparent because the overdue jobs with the maximum unit tardiness cost should be processed first to reduce the increase of tardiness cost in the system. Otherwise, the job will generate tardiness cost every second, and the tardiness cost is the largest compared with the tardiness cost generated by other jobs in *BF*.

(2) Select the job *j*, which has the minimum safe time *ST_j_*. Where *ST_j_* is calculated by Equation (15).

$$a_{2}=\arg\min_{j}\left(ST_{j}\right),\;j\in BF$$

*a*_2_ may be appropriate when no jobs in *BF* overdue or other actions do not have too many advantages.

(3) Select a job that has the shortest processing time (SPT).

$$a_{3}=\arg\min_{j}\left(\sum_{i=1}^{m}t_{ij}\right),\;j\in BF$$

(4) Select a job that has the longest processing time (LPT).

SPT and LPT are well-known dispatching rules and have been used as scheduling actions in [44,46,48].

$$a_{4}=\arg\max_{j}\left(\sum_{i=1}^{m}t_{ij}\right),\;j\in BF$$

(5) Select a job that obtains the maximum utilization rate *u_j_*′.

$$a_{5}=\arg\max_{j}\left({u_{j}}^{\prime }\right),\;j\in BF$$

*a*_5_ selects the most suitable job for current machine status, considering the utilization of machines. *a*_5_ may has larger priority when no jobs overdue or the $\psi_{j}$ has small standard deviation, etc.

The five scheduling actions above can provide efficient dispatching strategies under different production status. If more than one job in *BF* fulfills an action, a job is selected randomly among those candidate jobs. 

## 4. A2C

In this section, we adapt A2C to solve the dynamic PFSP with new job arrival. A2C is a DRL algorithm that equips with an actor-network $\pi_{\theta }$ and critic-network $V_{\phi }$ [62]. A2C selects an action with the probability $\pi_{\theta }(s_{t})$ generated by actor-network $\pi_{\theta }$, at state *s_t_*. The critic-network is used to estimate the state value of a state *s_t_* in the learning process. Recently, Liu, Chang and Tseng [47] used A2C to solve JSP to minimize makespan and achieved a good balance between makespan and execution time. However, A2C has not been used to solve the PFSP in currently published literature. We adapted the A2C to solve the dynamic PFSP, and the A2C-based training method are shown in Algorithm 1. In Algorithm 1, the new job arrival operation adds the newly arrived jobs during this step to *BF*. The operation is executed in the beginning and when a job is finished in *M*_1_. When a job is finished in *M*_1_, the *WIP* is updated by removing jobs that have already been finished in all machines.

The critic and actor networks are updated every *T* steps using gradients shown in lines 15 and 16 [62] in Algorithm 1. The *dr_t_* denotes the discounted reward of step *t* and is used as the target state value of step *t*. The estimated state value of step *t* is $V_{\phi }(s_{t})$. Thus, the gradient for critic-network is calculated by $\nabla_{\phi }{\left(dr_{t}-V_{\phi }(s_{t})\right)}^{2}$. For the actor-network, the difference between target and estimation state values is used to update the probability for selecting actions at state *s_t_*. Also, an entropy $H(\pi_{\theta })$, calculated by Equation(18), is considered to expand explorations.

(18) $$H(\pi_{\theta })=-\sum_{a_{t}\in A}\pi_{\theta }(a_{t}|s_{t})\log\pi_{\theta }(a_{t}|s_{t})$$

where *A* is the action space of the agent.

Algorithm 1 shows that an instance with *n* jobs is used to train the DRL agent for *EP* epochs, and the agent’s parameters are updated every *T* steps. Thus, the computation complexity of the training process for a single instance is O(*EP* × *n* × *T*). However, after training, the computation complexity for solving an instance is reduced to O(*n*).
**Algorithm 1.** The A2C-based training method.1:Initialize actor and critic network $\pi_{\theta }$, $V_{\phi }$2:**for** epoch = 1: *EP* **do**3:Perform new job arrival at time zero (current system time)4:Get current state *s_t_*5:**while***step* = 1: *n* **do** \\ *n* is the number of jobs for the selected instance6:Determine an action *a_t_* based on probability $\pi_{\theta }(s_{t})$ at state *s_t_*7:Select a job *j* from *BF* using action *a_t_*, process job *j* in all machines, obtain the finished time of job *j* in each machine8:Push forward the system time only to the time when job *j* is finished in *M*_1_9:Perform new job arrival and update *WIP* at current system time10:Get current state *s_t+_*_1_ and reward *r_t_*11:Store transition $\left\{s_{t},a_{t},r_{t},s_{t+1}\right\}$ of this step12:*s_t_* ← *s_t+_*_1_13:**if** step % *T* == 0 **then**14:Calculate discounted reward *dr_t_* of the *T* steps in reverse order using data in transitions, $dr_{t}=\left\{\begin{array}{c} r_{t}+\gamma V_{\phi }(s_{t+1}),\;\mathrm{for}\;\mathrm{the}\;T\mathrm{th}\;\mathrm{step}\\ r_{t}+\gamma dr_{t+1},\;\mathrm{for}\;\mathrm{the}\;\mathrm{first}\;T-1\;\mathrm{steps} \end{array}\right.$15:Update critic-network $V_{\phi }$ using gradient $\nabla_{\phi }{\left(dr_{t}-V_{\phi }(s_{t})\right)}^{2}$16:Update actor-network $\pi_{\theta }$ using gradient $\nabla_{\theta }\log\pi_{\theta }(a_{t}|s_{t})[dr_{t}-V_{\phi }(s_{t})]+\beta \nabla_{\theta }H(\pi_{\theta }(s_{t};\theta ))$17:**end if**18:**end while**19:**end for**

## 5. Numerical Experiments

In this section, a large range of instances is generated to train the A2C-based scheduling agent SCD. After training, the trained model of SCD is saved and used to provide intelligent decision-making of scheduling for dynamic PFSP. To evaluate the performance of A2C, we compared it with SDR, two other DRL algorithms, DQN and double DQN (DDQN), and two traditional meta-heuristics, IG and GA. We also tested the trained agent on a new set of extended instances with larger production configurations to verify the generalization of A2C.

A total number of 84 instances are generated for dynamic PFSP with new job arrival using different parameter settings of *n* and *m*, following [43,44]. The specific parameters for instances are listed in Table 1. As mentioned above, the due dates *d_j_* are determined by Equation (2). We noticed that when |*r*| < 10, the agent can be trained more efficiently. Based on this consideration, the *α_j_* is set.

The configuration of *n* and *m* in Table 1 is repeated three times. Hence, the total number of instances is 7 × 4 × 3 = 84. For each instance, three jobs are set as initial jobs, which arrive at time zero. Other jobs arrive following a poison distribution, which means the interval time of two successive arriving jobs follows an exponential distribution. The mean value of the exponential distribution is set as 30. To better evaluate the performance of the trained model, we divide the 84 instances as 59 (occupies 70% of all instances) training instances and 25 (occupies 30%) test instances randomly. In the training process, 3000 epochs are set. For each training epoch, all the 59 training instances are used to generate 59 episodes to train the scheduling agent. Thus, a total number of 59 × 3000 = 177,000 instances are used to train the agent. All instances, training and test results, and the video of solving dynamic PFSP using the trained agent and SDR are uploaded as Supplementary Materials.

All algorithms and workshop environments are coded with Python 3.8. The training and test experiments are performed on a PC with Intel(R) Core(TM) i7-6700 CPU @ 3.40 GHz CPU and 12 GB RAM.

### 5.1. Training Process of A2C

The A2C-based scheduling agent SCD is trained using the aforementioned training instances. Parameter settings for A2C are shown in Table 2. Note that the coefficient β is linearly decreased from 0.005 to 0.0005 during the first 70% epochs and remains at 0.0005 after the 2100th epoch.

For each epoch, the SCD is first trained on all of the 59 training instances and then tested on all of the 25 test instances. An instance is used to generate an episode under certain production configurations. Thus, each epoch contains 59 training episodes used to train the agent and 25 test episodes used to evaluate the agent’s performance after training at this epoch. The average total tardiness cost and episode reward on all test instances are recorded for each epoch. Note that the total tardiness cost in all experiments is rescaled by dividing 1000.

**Figure 2:**
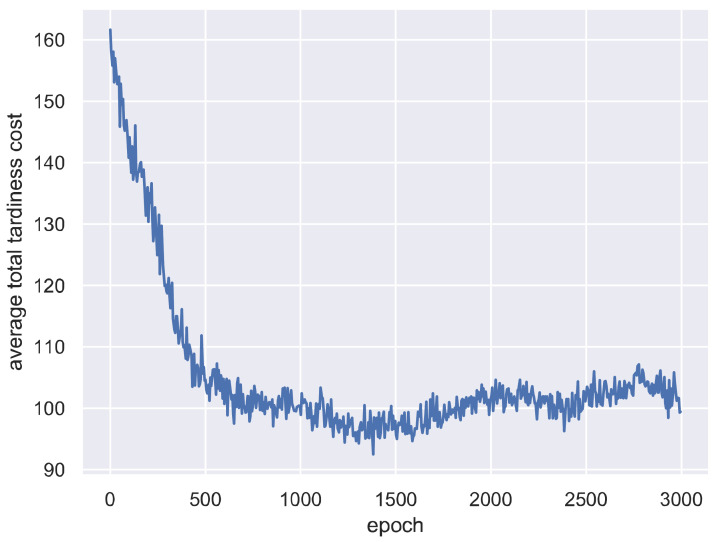
Average total tardiness cost on all test instances at each training epoch.

The average total tardiness cost on all test instances at each training epoch is shown in Figure 2. Figure 2 shows that the average total tardiness cost decreases dramatically with the increase of training epochs, meaning the SCD effectively learned to choose a good scheduling strategy and had a good generality for the unseen test instances. In particular, for the first 1400 epochs, the average total tardiness cost decreased from 163.55 to 91.88, which is a 43.82% improvement. The learning curve remains relatively stable after the 2000th epoch, even with some fluctuations. This indicates the SCD can provide reasonable scheduling solutions at a relatively stable level after training. Note that the solution seems not to keep at the global optimal found in the approximate 1300th epoch. This might be due to the exploration of A2C. The fluctuations and the problem of escaping from global optimal can be reduced by increasing training epochs.

**Figure 3:**
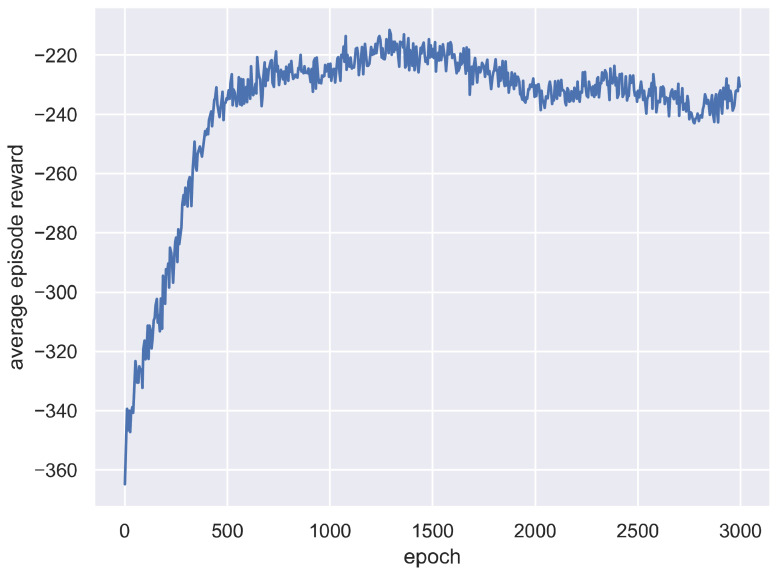
Average episode reward on all test instances at each training epoch.

The average episode reward obtained by SCD during the training epochs is shown in Figure 3, which can reflect the learning effect more directly. Figure 3 shows that the episode reward increases significantly with the increase of training epochs. Recall that a DRL agent learns to maximize the accumulated reward obtained during an episode. Thus, Figure 3 confirms the SCD indeed learned to perform better during the training process. The curve tendency of average episode reward is similar to that of average total tardiness cost, indicating the reward of SCD has a high correlation with the objective function of the studied scheduling problem, verifying the correctness of our reward design for SCD.

### 5.2. Comparison with SDR

**Figure 4:**
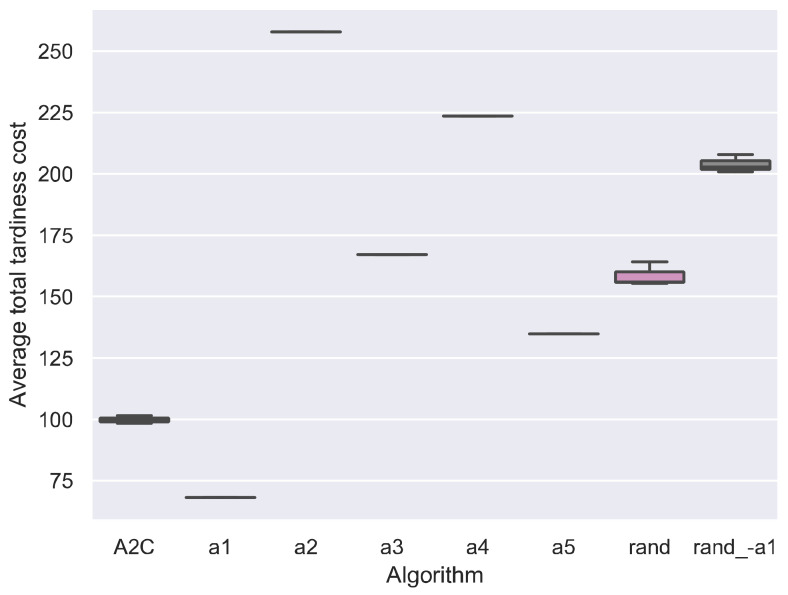
Comparison between A2C and SDR considering average total tardiness cost on all test instances.

Recall that the scheduling agent learns to choose the best scheduling action, i.e., SDR, at every rescheduling point. To evaluate whether the scheduling agent has learned to choose the best scheduling actions, we compared the A2C with SDR and random action strategys, as shown in Figure 4.

Figure 4 shows that the *a*_1_ obtains the best results. This may be because for the tardiness penalty criteria, always selecting the jobs with maximum current unit tardiness cost may be the best strategy, typically when the due date is too tight, and many jobs have waited in the buffer. It indicates that simple dispatching rules exist to generate high-quality solutions for the dynamic PFSP with total tardiness cost criteria. This kind of simple SDR is useful and efficient in practical production scheduling.

The results also indicate that the A2C fails to find the best actions at all rescheduling points. Between all actions, the *a*_1_ is the best one. However, in theory, there are situations when other actions are more appropriate than *a*_1_. Always selecting the *a*_1_ may not be the best strategy. Since the A2C is worse than *a*_1_, the DRL agent did not learn to choose the best actions at every rescheduling points. The trained DRL agent may not outperform its best pure action significantly when the performance of actions differs greatly. For example, the results in Lin, Deng, Chih and Chiu [46] show that the trained DRL agent MDQN only outperforms its best pure action MOPRN by 14.33%.

The random actions are also compared with the SDR strategy. Figure 4 shows that random action results are not the worst, but approximately the average of those of other actions. This may be because the random action is expected to generate a solution that equals the mean value of other actions’ solutions. To verify this conjecture, we tested the *rand_-a*_1_, the random action exclude *a*_1_. The results of *rand_-a*_1_ get worse when the best efficient action *a*_1_ is removed. Also, the *rand_-a*_1_ obtains the results close to the average results of other all actions exclude *a*_1_.

Compared between all actions, the *a*_1_ is the best one, followed by *a*_5_, i.e., select jobs with the maximum utilization rate *u_j_*′, and the worst one is *a*_2_, i.e., select the jobs with the minimum safe time *ST_j_*. Surprisingly, the *a*_2_ is worse than *a*_3_ (SPT) and *a*_4_ (LPT). This may be because the SPT and LPT are more related to the utilization, which is more important than the due date information influencing the safe time. The best action *a*_1_ outperforms the worst one *a*_2_ by 73.55%, which is a large scale. To improve the performance of DRL, more efficient actions should be designed, and less efficient actions are replaced. In addition, more efficient DRL algorithms should be proposed since the A2C fails to choose the best actions in every situation.

### 5.3. Comparison with DRL and Meta-Heuristics

To evaluate the performance of A2C, we compared it with two other widely used DRL algorithms, DQN and DDQN, and two traditional mate-heuristics, IG and GA.

#### 5.3.1. Training Process of DQN and DDQN

As mentioned above, the DQN has widely used to solve scheduling problems [44,46,49]. We adapt the DQN [63] and its variant DDQN [64] to solve the dynamic PFSP. The training procedure and instance settings are the same as those of A2C. After tuning, the hyper-parameters of DQN and DDQN are set. The learning rate for DQN and DDQN are $1\times 10^{-5}$ and $1\times 10^{-6}$, respectively. The memory size and batch size are 1000 and 32, respectively.

**Figure 5:**
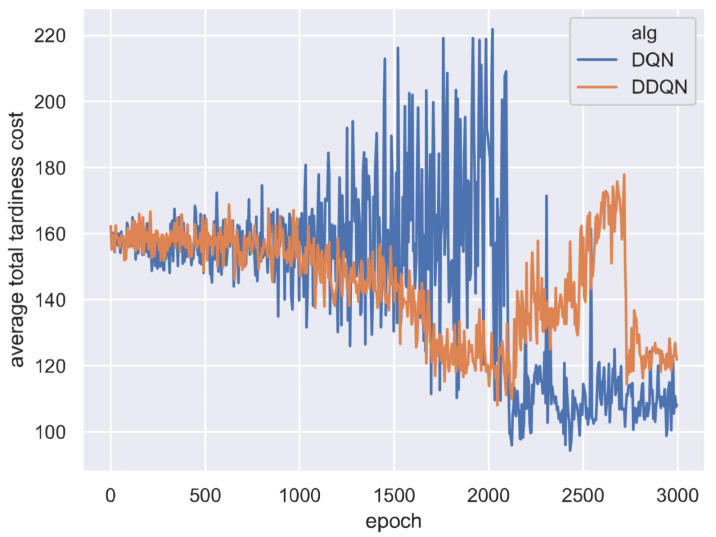
Average episode cost on all test instances at each training epoch for DQN and DDQN.

**Figure 6:**
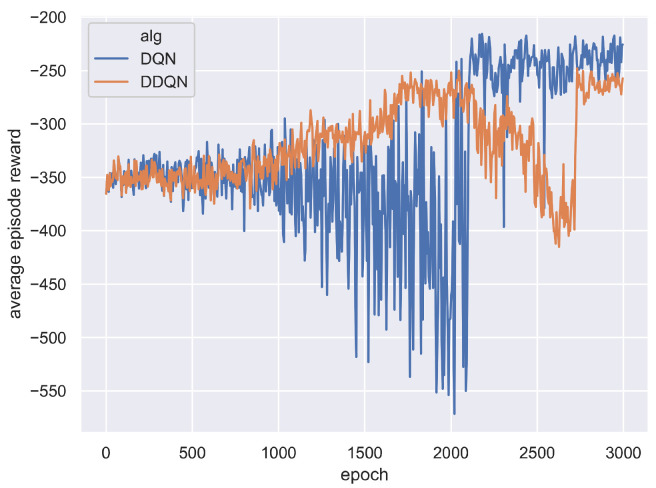
Average episode reward on all test instances at each training epoch for DQN and DDQN.

The training curves of DQN and DDQN are shown in Figure 5 and Figure 6. Figure 5 shows that the average total tardiness cost decreases during the training epochs, indicating the two DRL agents have learned to choose better actions at rescheduling points. Figure 6 shows that the average episode reward shows an increasing trend, verifying the agents indeed learned to generate better solutions. However, the learning curves of DQN and DDQN show a large fluctuation compared with those of A2C, indicating the A2C is easier to train. For the two DQN algorithms, the training curve of DDQN is smoother than those of DQN.

#### 5.3.2. IG and GA

To test the performance of A2C in solving dynamic PFSP, we also compared it with traditional meta-heuristics, which are extensively used in solving scheduling problems. From the review of [1], the IG-based algorithms show a very efficient performance compared with other meta-heuristics on the PFSP. In addition, GA is one of the most commonly used meta-heuristics in solving PFSP [1]. Thus, IG and GA are selected as traditional meta-heuristics to compare with our A2C-based scheduling approach.

For the dynamic PFSP, jobs arrive randomly. When a new job arrives, a rescheduling procedure is required to schedule the newly arrived jobs with jobs in the system. Following [16], we also set rescheduling times as 5 for an instance. When a rescheduling point is not reached, the newly arrived jobs are appended to the current job sequence to obtain a new job sequence. At each rescheduling point, the new job sequence is searched by meta-heuristics, i.e., IG and GA.

IG starts from an initial solution, executes destruction, reconstruction, local search, and acceptance criteria on the solution sequence for several iterations, and returns the optimized solution [21]. At the beginning of an instance, the initial solution *π_T_* is generated by putting all jobs arrived at time zero into a job sequence randomly. Jobs in *π_T_* are processed successively. When a rescheduling point is reached, the current job sequence *π_T_* is optimized through destruction, reconstruction, local search, and acceptance criteria for several iterations. In the destruction, |*π_T_*| × *p_desJ_* jobs are selected randomly from the current job sequence *π_T_* and removed from *π_T_* to the insertion job sequence *π_R_*. |*π_T_*|denotes the number of jobs in *π_T_*. After the destruction, the insertion job sequence *π_R_* is obtained, and the left job sequence in *π_T_* is denoted as *π_D_*. In the reconstruction, jobs in *π_R_* are selected randomly, inserted to all possible positions in *π_D_*, and finally placed in the position with minimum total tardiness cost to obtain a new job sequence. In the local search, the jobs in the current job sequence are randomly selected without replication. The selected jobs are then tested in all possible positions of the current job sequence and placed in the position with minimum total tardiness cost. When more than one position obtains the minimum total tardiness cost, one of those best positions is selected randomly. For the acceptance criteria, the aggravated solutions are generally accepted with a certain probability to expand searching scopes and escape from local optimal [58,65]. In this paper, worse solutions are accepted at a certain probability *τ*. The parameters of IG are tuned using the design of experiments (DOE) and analysis of variance (ANOVA) as performed in [58]. After tuning, the parameter settings of IG are *p_desJ_* = 0.2, *τ* = 0.05.

GA starts with an initial population, performs crossover, mutation, and selection between the population or individual for several iterations, and returns the optimized solution. Similar to IG, the initial solution is generated randomly using jobs arrived at time zero. At each rescheduling point, the current job sequence *π_T_* is permuted randomly to generate a population with *N* individuals. *N* = *min*(*N*′,|*π_T_*| × (|*π_T_*| − 1)), where *N*′ is the given population size. The population is evolved through crossover, mutation, and selection operations. In the crossover, the *N* individuals are paired randomly. Two individuals in a pair perform crossover with a probability *p_c_*. For the pairs that performs crossover, a crossover point is selected randomly. The sequences before the crossover point are switched between the two individuals in the same pair, and two new child job sequences are obtained. The repetition jobs in two child job sequences are replaced with jobs shown in the parent job sequence but not in the child’s sequence. In the mutation, every individual *l* in the population mutates with probability *p_m_*. If an individual mutates, two jobs in this individual’s job sequence are selected randomly and switched with each other. In the selection, *N* new individuals are selected from the current population with replication. Every individual will be selected at a probability *p_l_*, which is calculated by Equation (19).

(19) $$p_{l}=\frac{f_{l}}{\sum_{l=1}^{N}f_{l}},\;l=1,2,3,\ldots N$$

where *f_l_* is the fitness (total tardiness cost) of individual *l*. The parameters of GA are tuned using the aforementioned DOE and ANOVA. The parameter settings of GA are *N*′ = 50, *p_c_* = 0.80, *p_m_* = 0.10.

#### 5.3.3. Comparison with DQN, DDQN, IG, and GA

In this section, the A2C is compared with two other DRL algorithms, DQN and DDQN, and two meta-heuristics, IG and GA. After training, the trained models of all DRL agents are saved and used to generate scheduling actions during production.

**Figure 7:**
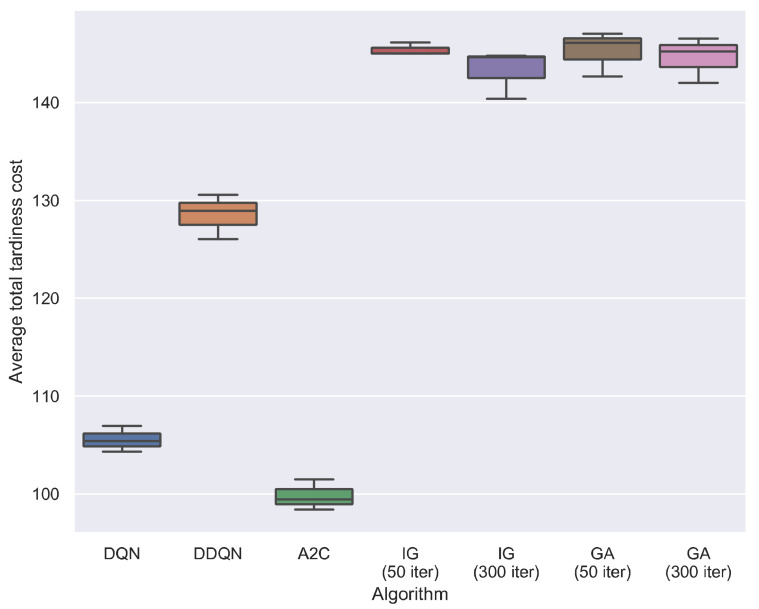
Average total tardiness cost of compared algorithms on all test instances. Three replications are carried out for each test instance. IG and GA are tested under two search iteration levels, 50 iterations and 300 iterations.

Figure 7 presents the average total tardiness cost of all compared algorithms on all test instances. As can be seen in Figure 7, A2C obtains the best results compared with all other algorithms. Typically, A2C outperforms two traditional meta-heuristics, IG and GA, by a large margin. We can also see that the three DRL algorithms outperform two meta-heuristics. This may result from the efficient scheduling action *a*_1_. One surprising result is that the DDQN is worse than DQN. This may be because the two algorithms are not fully convergence at the given epochs, and their results may change when more training epochs are given. For the two meta-heuristics, IG and GA are tested at two search iteration levels, 50 iterations and 300 iterations. When the iteration increased from 50 to 300, IG shows a significant statistical improvement, while GA does not. Overall, the two meta-heuristics with two search iteration levels do not differ significantly in the solution quality.

The specific average total tardiness cost and CPU times of the compared algorithms are provided in Table 3 and Table 4. Table 3 shows that the A2C and DQN generate the best results considering different production configurations. For the A2C, it outperforms IG and GA in solution quality and CPU times under all production configurations considered. Specifically, A2C, on average, outperforms IG and GA by 31.36% and 31.31% in solution quality, using only 0.04% and 1.35% CPU times that IG and GA spent when 50 searching iterations are provided for IG and GA. When IG and GA’s iterations increased to 300, A2C outperforms IG and GA by 30.34% and 30.98%, using merely 0.01% and 0.23% CPU times spent by IG and GA. For the two meta-heuristics, when iterations increased from 50 to 300, the CPU times increase by 279.71% and 493.01%, but IG and GA’s solution quality only improves slowly. IG obtains slightly better solution quality than GA, using more CPU times.

Table 3 and Table 4 further show that, as the production configuration *n* and *m* increases, the disparity in total tardiness cost between A2C and meta-heuristics increases. For example, A2C outperforms IG and GA with 50 iterations by 8.21% and 7.24%, when *m* is 5. However, when *m* is 20, A2C outperforms by 28.05% and 26.67%. This indicates that the A2C-based method has more advantages in solving large instances.

Note that the average CPU times of A2C for calculating a test instance is 0.21 s. Considering the number of jobs in test instances, the average decision time for taking a scheduling action is 2.16 ms. This is almost instantaneous and can be used in real-time scheduling.

The DRL method models the scheduling problems as an MDP where a DRL agent successively determines the job to be processed next [44]. The optimization of scheduling is realized by the successive decisions of DRL agents rather than by searching the job sequence directly as some meta-heuristics do. The generality of NN enables the DRL agents to behave well for unseen instances. In addition, the DRL agent can generate a scheduling decision instantly after trained. Thus, the DRL can be served as an alternative approach for dynamic scheduling problems.

### 5.4. Generalization to Larger Instances

To further verify the generalization ability of DRL, we tested the trained A2C agent on a novel set of extended instances with larger production configurations. This experiment aims to find out whether the performance deteriorates greatly when production settings different from the original training and test instances.

In the extended instances, the number of jobs *n* is increased from the original setting *n* = {20, 50, …, 200} to *n* = {300, 500, 800}. The number of machines *m* is set to 10. Other production settings are the same as the original instances. Each configuration of *n* and *m* are repeated three times. Thus, a total of 9 extended large instances are generated.

**Figure 8:**
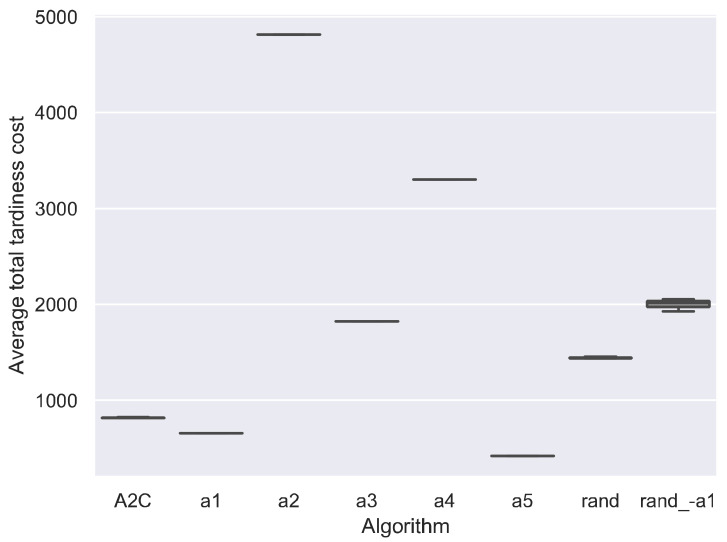
Comparison between A2C and SDR considering average total tardiness cost on extended instances.

The SDR is used as a baseline to evaluate whether the performance of A2C deteriorates when the production configuration becomes larger. The results of A2C and SDR on the extended instances are shown in Figure 8. Figure 8 shows that the A2C is worse than *a*_1_ and *a*_5_. The *a*_5_ becomes the best action, which may be because when too many jobs exist in the system, the utilization becomes more critical.

**Figure 9:**
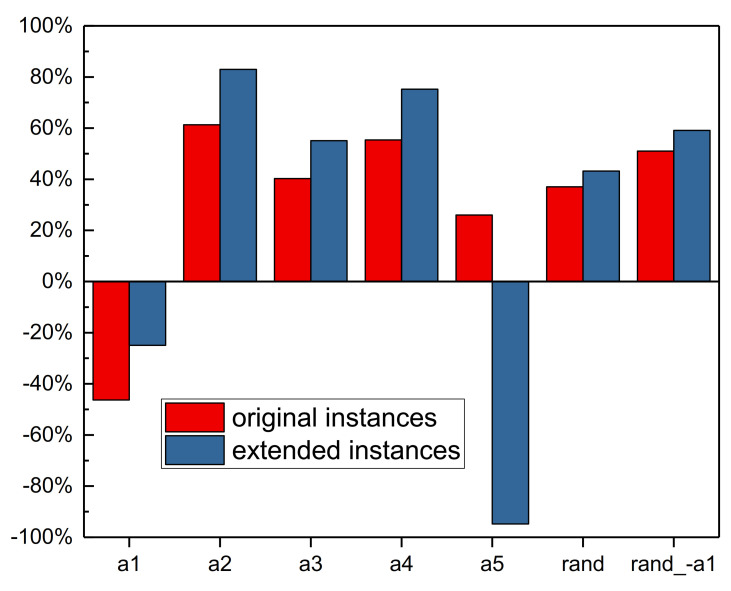
The percentages that A2C outperforms SDR considering average total tardiness cost on the original and extended instances.6. Conclusions.

To show the change of performance of A2C for larger production configurations, we provide the percentages that A2C outperforms SDR on the original and extended instances in Figure 9. As shown in Figure 9, the percentages on extended instances are larger than those on original instances for most SDR strategies except for the *a*_5_. This indicates that, on the whole, the DRL has a better generalization ability on larger production configurations compared with SDR.

## 6. Conclusions

This paper solved the dynamic PFSP with new job arrival to minimize total tardiness cost using DRL. This study aims to establish an intelligent decision-making scheduling system to provide real-time optimization for dynamic scheduling problems. The whole procedure of solving dynamic PFSP using DRL is illustrated, and the mathematical model is established. The DRL-based scheduling system is proposed with state features, actions, and reward designed for the scheduling agent and workshop environment. Five scheduling actions are designed to perform well at different production states. The state features with 17 dimensions are designed to provide directly related information for choosing scheduling actions. The A2C algorithm is adapted to train the scheduling agent SCD to learn an appropriate scheduling action at different states. A total of 84 instances are generated to train the A2C-based scheduling agent, and extensive comparisons are carried out. The trained scheduling agent is compared with SDR, two other DRL algorithms, DQN and DDQN, two traditional meta-heuristics, IG and GA. The training curve shows that the average total tardiness cost decreases significantly with training epochs increases, indicating the scheduling agent has learned to choose appropriate scheduling actions. The comparison experiments show our A2C-based scheduling agent performs best among all compared DRL and meta-heuristics. Typically, the A2C-based scheduling agent outperforms IG and GA by a large margin, both in solution quality and CPU times. Our A2C-based scheduling agent can generate a scheduling action in 2.16 ms on average. This can be used for real-time scheduling and online decision-making in a real production workshop. In addition, the A2C-based scheduling agent shows well generalization on unseen instances with larger production configurations. The scheduling agent can still be trained during production. Our study contributes to establishing a self-organizing and self-learning scheduling system.

Future research can consider more realistic characteristics, such as machine breakdown, transportation time, etc., and solve real-time scheduling of more complex workshops, such as reconfiguration workshop, hybrid flowshop, and distributed workshop. In addition, more studies can be applied to design more effective state features, action spaces, and reward for the scheduling system. Moreover, more DRL algorithms could be studied to improve the scheduling agent’s learning effect and solution performance.

## Figures and Tables

**Table 1:** Parameters for instances.

Parameter	Value
Number of jobs (*n*)	{20, 50, 80, 100, 120, 150, 200}
Number of machines (*m*)	{5, 10, 15, 20}
Number of initial jobs for every instance	3
Interval time of job arriving	E(1/30)
Processing time on a machine (*t_ij_*)	U[1, 100]
Unit tardiness cost of a job (*α_j_*)	U[0, 5]

**Table 2:** Parameter settings for the training process and DRL algorithms.

Parameter	Value
Number of hidden layers	3
Number of neurons in each hidden layer	100
Number training epochs (*EP*)	3000
Discount factor (*γ*)	0.98
Update step iteration (*T*)	5
Learning rate of actor and critic	$2\times 10^{-6}$, $5\times 10^{-6}$
Range of entropy coefficient (β)	0.005~0.0005

**Table 3:** Average total tardiness cost grouped by the number of jobs *n* and machines *m* for compared DRL and meta-heuristics. IG and GA are tested under two search iteration levels, i.e., 50 iterations and 300 iterations. The best results are highlighted in bold.

		DRL			Meta-Heuristics			
					50 Iterations		300 Iterations	
		DQN	DDQN	A2C	IG	GA	IG	GA
***n***	20	**13.88**	21.52	16.06	19.34	17.68	19.16	19.67
	50	50.09	65.61	**48.98**	75.43	75.83	73.38	77.13
	80	94.76	83.05	**60.79**	114.45	112.9	115.62	112.69
	100	**101.31**	143.59	108.57	161.13	159.64	161.99	165.87
	120	152.35	208.83	**144.45**	205.15	204.78	201.4	207.9
	150	175.06	190.7	**163.28**	222.84	213.67	220.35	211.7
	200	**150.53**	172.42	197.99	255.62	256.19	282.16	246.4
***m***	5	**43.76**	63.19	46.76	50.94	50.04	50.41	49.21
	10	**73.73**	78.09	74.81	101.02	100.75	106.84	101.21
	15	113.44	191.28	**106.21**	195.85	193.4	192.31	195.48
	20	265.5	249.54	**232.93**	323.72	316.15	317.65	321.87
**Ave**		105.55	128.52	**99.79**	145.39	145.28	143.25	144.58

**Table 4:** Average CPU times (s) grouped by the number of jobs *n* and machines *m* for DRL and meta-heuristics. IG and GA are tested under two search iteration levels, i.e., 50 iterations and 300 iterations. The CPU time for an instance is the total CPU time required for solving that instance.

		DRL			Meta-Heuristics			
					50 Iterations		300 Iterations	
		DQN	DDQN	A2C	IG	GA	IG	GA
***n***	20	0.03	0.03	**0.02**	12.8	56.62	4.35	32.45
	50	0.09	0.09	**0.07**	63.94	230.29	10.31	54.08
	80	0.19	0.18	**0.15**	63.2	278.19	6.39	37.85
	100	0.27	0.26	**0.21**	337.07	1301.06	15.84	94.28
	120	0.33	0.34	**0.27**	751.39	2791.67	25.53	121.65
	150	0.49	0.49	**0.38**	1020.36	3909.1	21.25	165.13
	200	0.68	0.69	**0.49**	3441.1	13023.42	34.01	200.35
***m***	5	0.17	0.16	**0.14**	246.99	914.52	10.82	70.21
	10	0.24	0.23	**0.18**	594.53	2258.63	13.82	81.65
	15	0.31	0.31	**0.24**	514.63	1665.1	17.72	89.91
	20	0.41	0.42	**0.33**	900.46	3891.24	24.32	156.79
**Ave**		0.26	0.26	**0.21**	526.99	15.59	2001	92.45

## Data Availability

Not applicable.

## Supplementary Materials

All instances, training and test results, and the video of solving dynamic PFSP using the trained agent and SDR are available at https://osf.io/tynrd/?view_only=bbea6a78dff042e3a5952c3283031676.
